# Co‐Production and Implementation of ‘Count Me In’: A Bottom‐Up Approach to Inclusive Research and Participation in a National Health Service in England

**DOI:** 10.1111/hex.70326

**Published:** 2025-06-23

**Authors:** Oladayo Bifarin, Jahanara Miah, Michelle Harvey, Gail Faragher, Jade Thai, Dennis Dewar, Jenny Garden, Lindsey Nicholson, Nicola Wilson, Nusrat Husain, Dan W. Joyce

**Affiliations:** ^1^ Mersey Care NHS Foundation Trust, Mental Health Research for Innovation Centre, NHS Informatics Merseyside Liverpool UK; ^2^ School of Nursing and Advanced Practice Liverpool John Moores University Liverpool UK; ^3^ Senior Research Leader Programme, National Institute for Health and Care Research (NIHR) London UK; ^4^ School of Health Sciences, Faculty of Biology, Medicine and Health The University of Manchester Manchester UK; ^5^ Department of Primary Care and Mental Health and Civic Health Innovation Labs University of Liverpool Liverpool UK

**Keywords:** co‐production, Count Me In, health equity, inclusive research, mental health research, NHS, ‘opt‐out’ recruitment, patient engagement, PPIE

## Abstract

**Background:**

Research‐active National Health Service (NHS) services are linked to better care quality and health outcomes. However, traditional research participant recruitment methods, such as ‘opt‐in’ strategies, often face challenges in reaching diverse populations. The ‘Count Me In’ (CMI) system was introduced to address these barriers through an ‘opt‐out’ model, aiming to normalise research participation and promote inclusivity. At Mersey Care NHS Foundation Trust, a bottom‐up approach was employed to adapt CMI, ensuring meaningful engagement with service users, carers and communities in its design and implementation.

**Methods:**

CMI was co‐produced with stakeholders through a series of workshops, discussion groups and consultations with over 300 participants, including service users, carers and NHS staff. Key activities included listening exercises to understand concerns, co‐designing campaign materials and forming a Patient and Public Involvement and Engagement (PPIE) Advisory Group. The group provided ongoing guidance to ensure that the system aligned with the needs of underserved communities and upheld ethical and cultural sensitivity.

**Findings:**

Stakeholders widely supported the ‘opt‐out’ approach, recognising its potential to improve research inclusivity. Participants highlighted the importance of clear communication, cultural sensitivity and robust data protection measures. Specific research priorities, such as mental health and social isolation, were identified. Co‐produced materials, including plain‐language guides and culturally appropriate visuals, addressed concerns about privacy, stigma and accessibility, fostering trust and confidence in the system.

**Conclusion:**

The CMI system is an acceptable and scalable model for inclusive research recruitment, embedding research into routine care. The bottom‐up approach ensured the system was tailored to local needs, promoting equity and accessibility.

**Patient and Public Contribution:**

A partnership approach working with PPIE leads at the Mental Health Research for Innovation Centre (M‐RIC) ensured that service users, carers and community members shaped the CMI system through extensive co‐production activities. The development of the system, therefore, reflected their lived experiences and priorities, thereby enhancing its inclusivity and impact.

## 1 Introduction

Healthcare research is beneficial in improving care quality and outcomes for people and service users. However, traditional recruitment methods for participation in clinical and research trials often fail to reach underserved populations, compounding the disparities in health outcomes. This issue is particularly evident in mental health research, due to various factors including systemic barriers, cultural stigma and reliance on clinician‐led approaches, limiting inclusivity in research [1]. The ‘Count Me In’ (CMI) system [2] aims to address these challenges by implementing an ‘opt‐out’ recruitment model that normalises research participation as part of routine care. CMI aims to democratise mental healthcare research by reducing barriers to access and offering opportunities for any individual to participate in research and benefit from advancements in healthcare [3]. Co‐producing such initiatives with service users, carers and communities is imperative so the CMI process reflects lived experiences, addresses specific concerns and builds trust [4, 5, 6].

This paper therefore explores the co‐production and implementation of the CMI system within Mersey Care NHS Foundation Trust, aiming to enhance inclusivity in research participation through an ‘opt‐out’ model. By engaging service users, carers and NHS staff in its design, the initiative seeks to address barriers to participation, particularly among underserved groups. The paper outlines the bottom‐up approach used to adapt CMI to local needs, ensuring ethical, transparent and community‐driven research engagement.

## 2 Background

Clinical services within the National Health Service (NHS) that engage in research activities are linked to reduced mortality rates, especially as NHS organisations that conduct research demonstrate improved quality of care and better health outcomes [7, 8]. Other than direct impact generated through clinical trials, creating an enabling research environment within services has been evidenced to foster sustained engagement and motivation among service users to participate in and remain committed to research endeavours [9]. Despite these advantages, recruiting participants for clinical research presents substantial challenges [10]. Specifically, recent efforts to improve research inclusivity within the NHS have examined various approaches to patient engagement [3]. Traditional ‘opt‐in’ methods, which require service users to explicitly indicate their willingness to participate in research, have experienced limited success due to low participation rates [10]. Further, excessive reliance on clinician‐led recruitment strategies can introduce gatekeeping and often results in biases and uneven access to research opportunities [2, 3].

Addressing this, the CMI system was introduced in August 2021 by the Oxford Health NHS Foundation Trust [3]. CMI adopts an ‘opt‐out’ strategy, enabling service users to be informed of research participation they may be eligible for, unless they decline (‘opt out’), significantly diversifying the pool of potential participants. Henshall, Potts, Walker, Hancock, Underwood, Broughton, Ede, Kernot, O'Neill, Geddes and Cipriani [11] showed that approximately 80.2% of surveyed staff and members favoured adopting an ‘opt‐out’ approach over the existing ‘opt‐in’ system and only 11% of service users had participated in a research discussion with their clinician under the ‘opt‐in’ system. Similarly, the ‘opt‐out’ option afforded under‐represented or marginalised groups to be aware of and participate in clinical research, making the participation more equitable.

The ambition is to expand the CMI system across other NHS organisations, integrating research into standard patient care and fostering inclusivity by guaranteeing that all service users, irrespective of their diagnosis or familiarity with clinicians, are presented with opportunities to be informed of research participation relevant to them [3]. Mersey Care NHS Foundation Trust is one of the largest providers of community and mental health services in England, with a different research infrastructure as compared to Oxford Health NHS Foundation Trust. While Oxford Health benefits from research infrastructure including Biomedical Research Centres (BRCs), we saw an opportunity to implement CMI in a way that complements our unique organisational context and strengths.

Consequently, we decided to adopt a ‘bottom‐up’ approach to implementing CMI, which involved engagement at every organisational level, where plans are formulated at the lower tiers and progressively channelled upwards through successive layers until they are reviewed and finalised by senior management. We took this stance in response to implementation challenges highlighted by Henshall, Potts, Walker, Hancock, Underwood, Broughton, Ede, Kernot, O'Neill, Geddes and Cipriani [11] with a specific focus on careful consideration of data protection regulations and communications plan, honing the purposes and processes of the ‘opt‐out’ system to avoid confusion and misinterpretation.

## 3 Methods

In September 2023, the Board of Executive Directors at Mersey Care NHS Foundation Trust unanimously approved the development of the CMI system, adapted to local cultures of practice and residents' needs, in recognition of a need to bolster the Trust's commitment to research and innovation (R&I). Between April 2024 and September 2024, the implementation team adopted a bottom‐up, phased approach to engage with stakeholders, ensuring the initiative is implemented in a way that fosters a sense of ownership and control among those involved. This has included listening exercises, such as workshops, gathering input from staff, service users and community members, as well as building partnerships with patient groups and advocacy organisations to co‐design strategies that meet their needs. The CMI system has been implemented through a phased approach, beginning with targeted community engagement, followed by co‐design of communications, and later scaling into formal system‐wide adoption with continuous feedback loops. Leadership in the Trust has been encouraged at all levels, with frontline teams and service users taking active roles in shaping a localised version of the CMI system.

### 3.1 Theoretical Framework

This study is underpinned by social constructivism [12], which views knowledge as co‐created through social interactions and shaped by cultural and contextual factors. It reflects a subjectivist epistemology and a relativist ontology, recognising that equity in mental health care is socially situated and influenced by lived experiences. We adopted the Ladder of Engagement Framework [13], focusing our engagement on devolving, collaborating, involving and informing. These approaches amplify community voices, ensuring their lived experiences directly shape decisions on CMI's implementation. By prioritising inclusive and meaningful engagement, we aimed to address inequities in mental healthcare and support.

### 3.2 Ethical Considerations

As our work is centred upon Patient and Public Involvement and Engagement (PPIE), formal ethical approval was not required. Nonetheless, we adhered to key ethical principles, including autonomy, non‐maleficence, beneficence and justice [14, 15, 16], ensuring our approach was inclusive, equitable and transparent. We embraced autonomy and non‐maleficence by valuing diverse perspectives and providing clear, accessible and adoptable sensitive approaches to support informed participation. Beneficence was reflected in creating a supportive environment where participants felt safe and empowered to share their insights. To uphold justice, we prioritised the inclusion of marginalised voices, addressing barriers such as language or access to ensure equitable participation. By embedding these principles into our work, we ensured that diverse voices were not only included but central to shaping meaningful outcomes, reflecting our commitment to equity and inclusion.

### 3.3 Procedure

#### 3.3.1 Phase 1: Engagement With Stakeholders

We organised initial engagement workshops with community organisations and groups; these workshops were designed to gather input from various community members, ensuring that different voices, such as residents, local organisations and marginalised groups in Liverpool are represented (See Table 1):

**Table 1:** Engagement workshops with staff, service users and local communities.

Groups	Nature of group	Number of participants
Knowsley older persons' coffee morning	Older adults, including people with dementia and carers; male and female participants.	11
Mersey Care and Health Watch Professionals meeting	Health professionals; male and female.	10
South Asian Women's Group	Bangladeshi, Pakistani and Indian women from the local community in Liverpool.	10
M‐RIC Public Advisors workshop	Mixed group of service users, carers and members of community organisations; male and female.	25
Speak up‐learning disability group	Individuals with learning disabilities and their carers; male and female participants.	20
Mersey Care NHS Foundation Trust ‐ Mental health and well‐being practitioners	Mental health and well‐being practitioners across services.	13
Mary Seacole House‐Cantonese‐speaking group	Male and female participants originally from Hong Kong; Cantonese‐speaking.	15
Mary Seacole House‐ethnic minority/asylum seeker group	Mixed group including Iranian, Syrian, Congolese, Kenyan, Sierra Leonean, French, Spanish and Mexican participants; male and female.	13
The Life Rooms Bootle	Service users and carers.	4
Mersey Care Council of Governors	A mix of service users, carers and members of the public providing input at the governance level.	30
Mersey Care Staff online ‘Your Brief’	Healthcare staff, including clinicians, Psychological Wellbeing Practitioners (PWPs) and other roles.	129
Count Me In Information Events (face‐to‐face and online)	Member of the public in Liverpool.	51
Total		331

As part of our intention to engage stakeholders in the development of the CMI system, we required feedback from service users, carers and health professionals on various key viewpoints. The consultation workshops provided information about the CMI system and then asked a series of questions to the stakeholders to gather their insights and address potential concerns. The feedback schedule, co‐produced with the lead service user and Mental Health Research for Innovation Centre (M‐RIC) PPIE team, focused on the following key questions (See Table 2):

**Table 2:** Feedback question for the CMI system.

Questions	Rationale
1. What do you think about Count Me In‐and being contacted about research	We asked service users, carers and staff for their thoughts on the CMI system, particularly how they felt about the idea of being contacted regarding research opportunities. Understanding their viewpoints was important in determining the acceptability of this new approach.
2. What concerns do you have?	Participants were invited to share any apprehensions they might have about the CMI system. These included concerns related to privacy, data security, the frequency of contact, and any potential impact on their care experience.
3. What benefits can you see happening once researchers are able to contact all service users?	We explored potential benefits they anticipated from supporting researchers to contact all eligible service users. This involved discussions on how increased research participation could lead to improved treatments and improved mental health services.
4. Is there anything you think we should be researching?	We also asked for input on specific areas of research they believed should be prioritised. This ensured that the CMI system would align with the interests and needs of the service users and staff, making the research more relevant and impactful.

The groups listed in Table 1 were engaged using the questions and prompts outlined in Table 2. The insights gained from these conversations informed the actions and design adaptations captured in Tables 3 and 4, illustrating a clear flow from consultation to implementation.

**Table 3:** Additional themes with ethnic minorities and refugee groups.

Theme	Key issues
Confusion and Uncertainty	Participants expressed confusion about research procedures, purpose and expectations.
Curiosity and Interest	Participants showed interest in the CMI system and its potential benefits to health and well‐being.
Cultural Hesitancy and Stigma	Cultural differences and mental health stigma discouraged participation due to fear of judgement within communities.
Need for Accessible Information	Participants emphasised the need for materials in multiple languages and formats, including face‐to‐face explanations.
Privacy and Confidentiality Concerns	Participants feared data misuse and potential negative impacts on their personal lives or health treatment.

### 3.4 Findings

Themes From Engagement Workshops

Aligned with the key questions developed for the engagement workshops, two facilitators took notes on key points raised by participants, in addition to ideas and concerns raised. These notes were summarised to ensure that the main themes and participant perspectives were accurately captured. The summarised notes were then reviewed and compiled into a central document, where common topics and patterns began to emerge. These recurring themes were grouped into broad categories to guide further development of the CMI system. The responses are themed below.

#### 3.4.1 Positive Acceptance and Support for Count Me In

There was an overwhelmingly positive response to the CMI system. Participants consistently viewed CMI as valuable, highlighting its potential to improve inclusivity and engagement in research across diverse and underserved groups.

#### 3.4.2 Communication Preferences and Concerns

Participants were open to being contacted about CMI but raised concerns about communication approaches, highlighting the need for communication options adapted to different communication preferences with tailored and accessible information, especially for vulnerable groups, such as those with dementia, complex mental health conditions and learning disabilities.

#### 3.4.3 Desire for Support and Accompaniment

Preference for additional support during the research process was highlighted, and participants articulated the importance of family members or carers being included in communications, alongside involvement in the decision‐making processes.

#### 3.4.4 Research Ideas and Priorities

Participants suggested a range of research ideas and priorities, including the impact of social isolation on mental health and the accessibility of mental healthcare for individuals with intellectual disabilities.

#### 3.4.5 Awareness of Research

There was a general acknowledgement of the value of CMI as a means of staying informed about research and how earlier engagement in research could be beneficial, indicating a desire for timely inclusion.

#### 3.4.6 Concerns and Hesitations

Concerns were raised linked to research gaps in engaging with learning disability groups. Hesitancy about the research process, along with stigma in participating in mental health research, was a key concern and echoed prominently by ethnic minority groups, along with uncertainty about decision‐making criteria. Concerns raised was particularly for groups who may lack capacity to consent due to cognitive decline, dementia, complex mental health challenges and intellectual disabilities. Building trust between researchers and participants, particularly around decision‐making was deemed pertinent for sustained engagement.

#### 3.4.7 Informed Consent and Data Quality

Participants stressed the importance of clear informed consent processes for when actual research opportunities arise and upholding high standards for data quality therein. Concerns were raised about researcher bias in terms of how researchers may interpret data to support their predetermined ideas and selectively analyse data to fit their ideas. The need to ensure participant autonomy was repeatedly mentioned, highlighting the ethical dimensions of research participation.

#### 3.4.8 Logistics and Infrastructure

Participants highlighted the need for clarity regarding CMI planning, including communication methods, infrastructure and accessibility. Past negative experiences with research were mentioned by a few participants, pointing to the need for a more inclusive and accessible approach in promoting and organising research activities across the Trust.

#### 3.4.9 Building Trust and Co‐Production

Trust‐building between researchers and participants as a key factor for successful engagement. The need for co‐production was therefore identified as an essential practice, with participants stressing the importance of research that is collaboratively developed to meet their specific needs and concerns.

#### 3.4.10 Minoritised Ethnic Groups and Refugee Groups

In addition to the above themes, additional themes identified from the engagement workshop with minoritised ethnic and refugee groups are highlighted in Table 3. As this was an implementation‐led initiative rather than a research study, we did not collect demographic data in a formal or identifiable manner. However, we were deliberate with our approach with communities facing systemic marginalisation, including South Asian, Chinese and Black populations, guided by partnerships with trusted local organisations.

**Figure 1:**
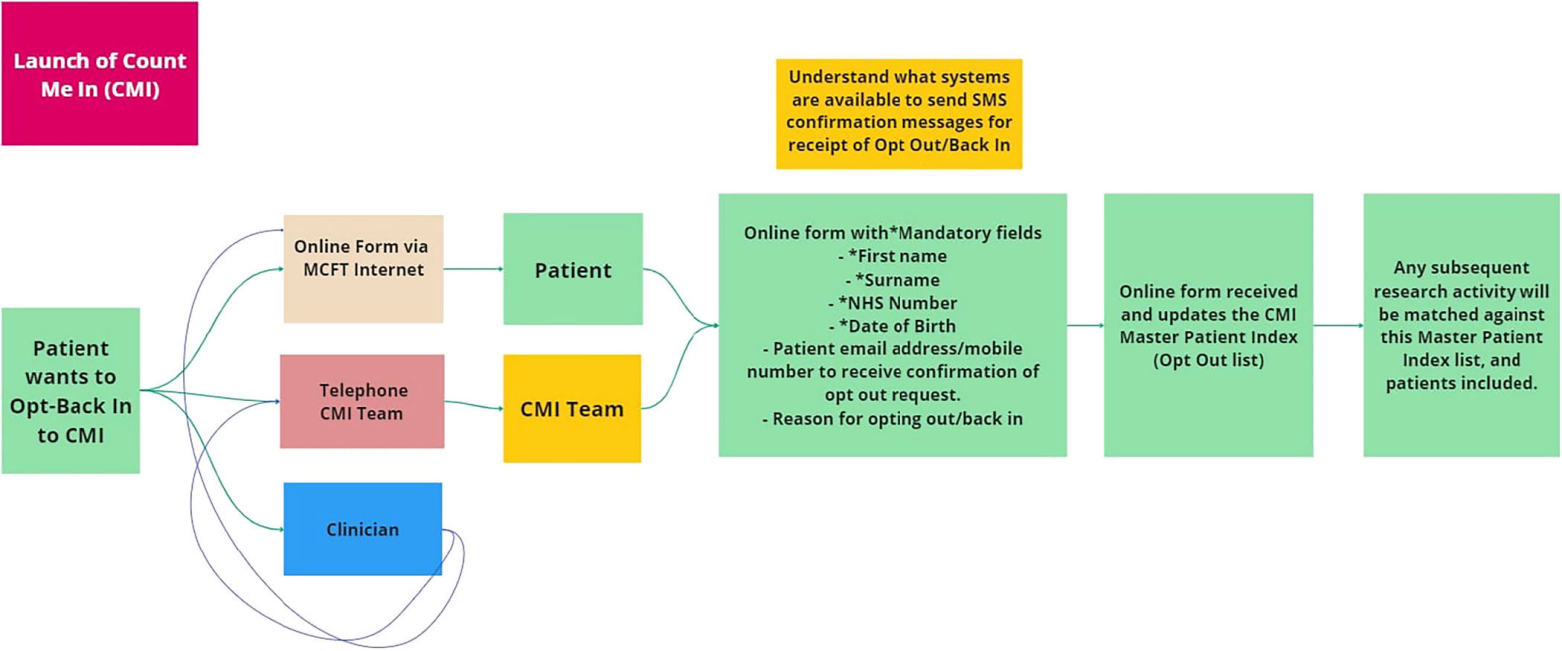
‘Opt‐back in’ flow chart Patient and Public Involvement and Engagement (PPIE): empowering voices.

In response to stakeholders, enhancements such as visual flowcharts have been introduced to clarify CMI processes further (see Figure 1). These improvements demonstrate our ongoing commitment to addressing concerns and ensuring trust and confidence in the system.

### 3.5 Phase 2: Socialised Power in Action

Following the initial individual workshops, all participants from the engagement workshops were invited to join a larger group focused on the co‐production of the CMI campaign materials; 20 participants expressed interest in participating. This co‐production group was established to ensure that the development of the campaign was informed by the collective insights and experiences of a diverse range of participants, including service users, carers and staff members.

#### 3.5.1 Co‐Production of the CMI Campaign Materials

The co‐production workshop for the CMI campaign materials brought together 20 participants, creating an inclusive space for service users, carers, clinical staff members and researchers. The event began with a co‐produced stage drama performance by service users and carers that employed improvisation to develop an imagined conversation between researchers and patients. The presentation engaged the audience through an innovative, engaging approach to communicate the core objectives of the CMI system. Following the performance, participants were provided with a brief overview of the background and context of CMI, including the rationale for its implementation. Key findings from engagement workshops (presented above) were also presented, ensuring that the workshop was grounded in the perspectives and insights already gathered from stakeholders. Through structured discussions and activities, participants played an active role in shaping the communication strategies and design concepts for CMI. Below is an outline of participants' contributions to the development process:

Participants were asked to assess the readability of the campaign materials; they were provided with draft versions and asked to rate the clarity of the main messages. Specifically, they reviewed how well the materials communicated key information and whether the message was easily understandable by a broad audience. Participants provided comments on how clear the main messaging was, pointing out any areas that required further refinement to ensure simple messaging of CMI information. The group was encouraged to highlight any sections of the material that they found confusing or unclear, offering suggestions to make the information more understandable.

**Figure 2:**
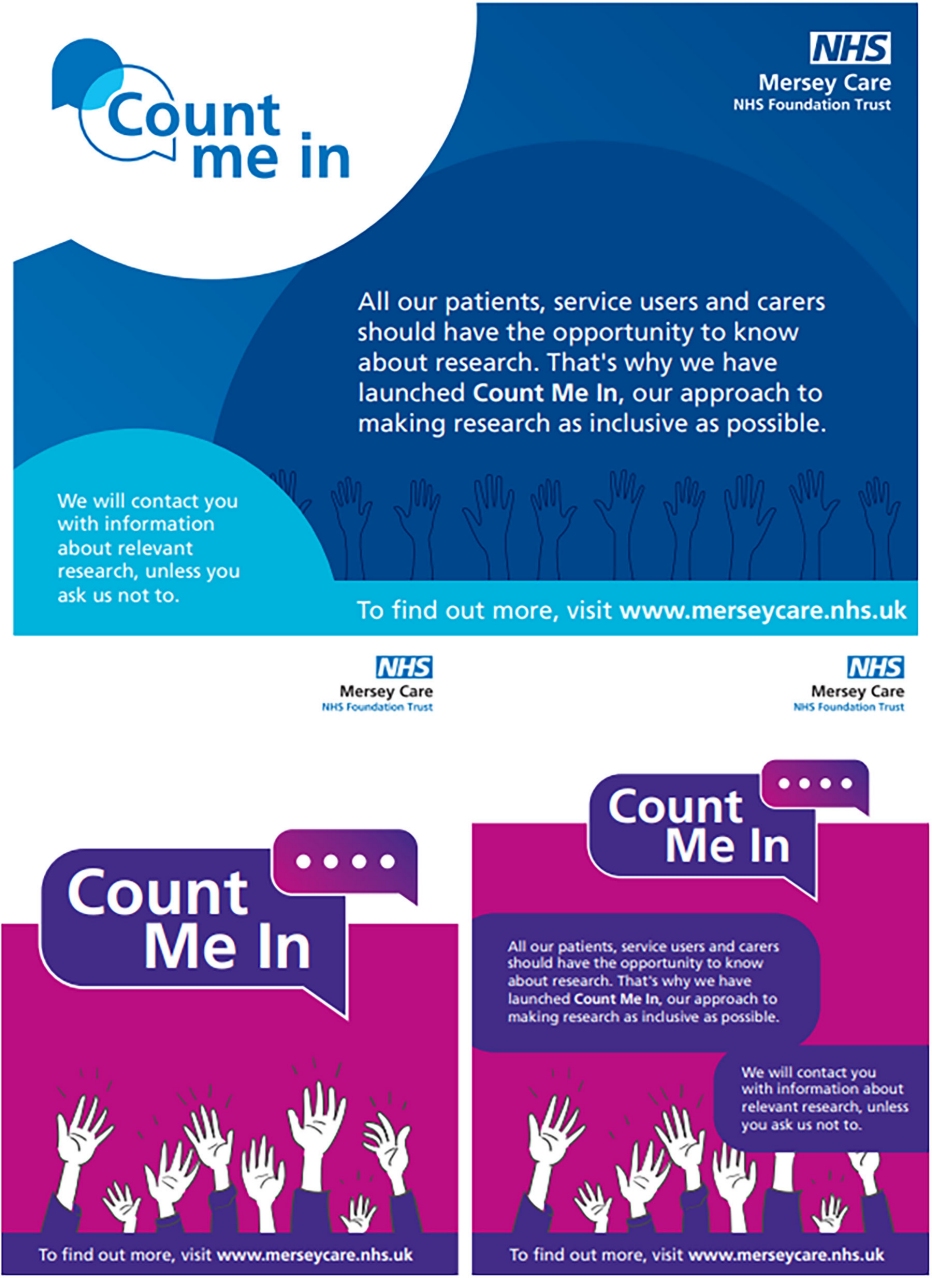
Early‐stage CMI campaign material design concepts.

Ensuring that the campaign materials were accessible to diverse audiences was a priority. Participants were asked to assess the ease of understanding the language used and the inclusivity of representations in visual elements. Feedback was sought on whether the language used was accessible, particularly for individuals who might not understand research or healthcare terminology. Participants were asked to pinpoint any terms or phrases they found difficult to understand, with the aim of ensuring that the material was free from technical jargon and easily understandable for all audiences. The group also reflected on whether the instructions for next steps or calls to action were clear and easy to follow, ensuring that participants would know exactly how service users could engage with CMI and especially, the ‘opt out’ process. Participants were encouraged to offer specific suggestions for improving the content, language or design of the materials. Based on the feedback, the changes were made and shared with the CMI PPIE Advisory Group for final approval of the campaign materials. An example of the early‐stage CMI design concepts (see Figure 2 and Supporting Information), which the CMI PPIE Advisory Group provided feedback on.

**Figure 3:**
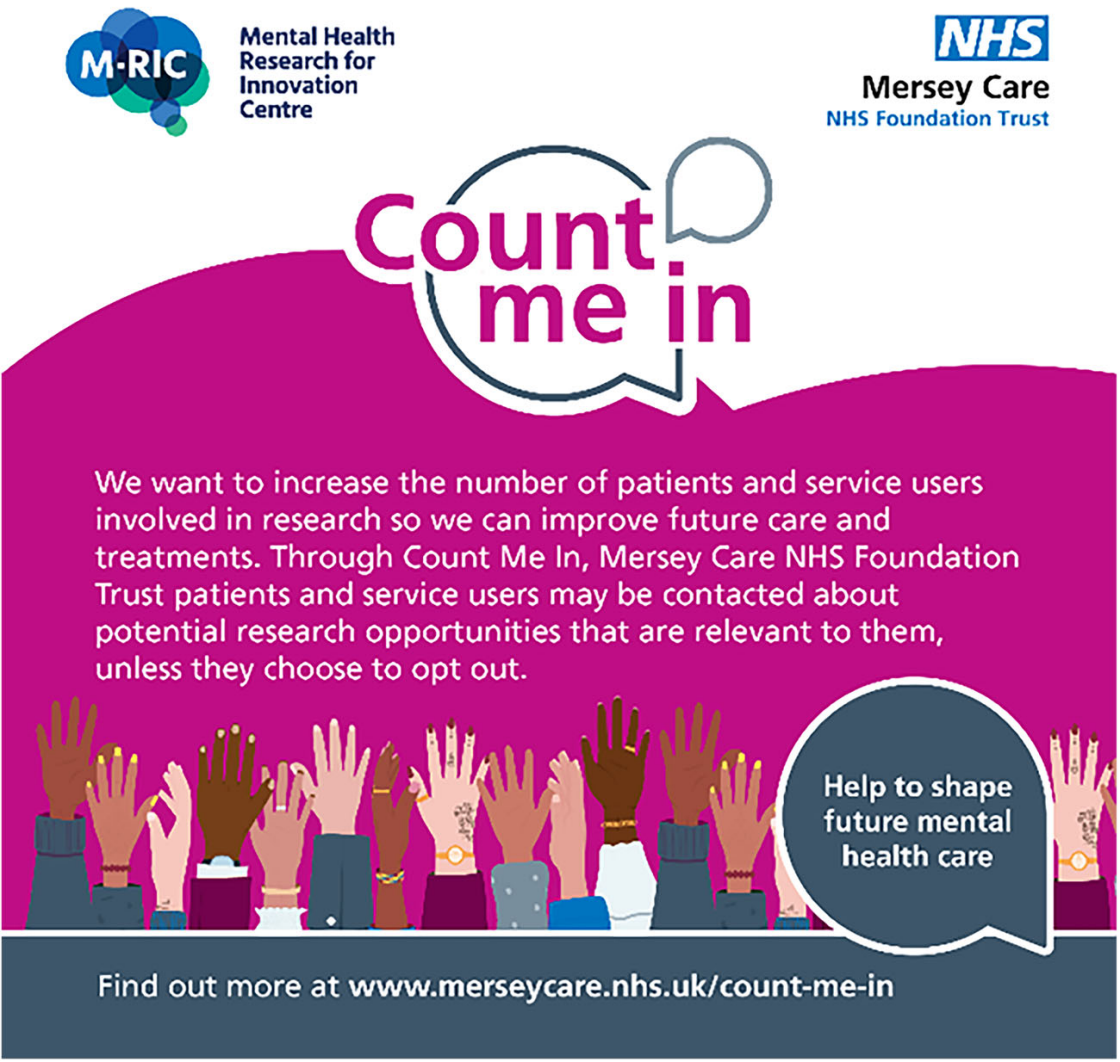
Final CMI design as a social media tile.

The CMI PPIE Advisory Group provided feedback on the above design concepts. Examples of their responses include confusion around the four dots in Figure 2 and advice to emphasise the inclusivity of the hand design and to ensure the accessibility of the colours used. The co‐produced revised version is shown as Figure 3.

#### 3.5.2 CMI PPIE Advisory Group

A CMI PPIE Advisory Group was established with the purpose of co‐producing key aspects of the project, including communications, information governance and further PPIE activities.

#### 3.5.3 Recruitment and Structure

The CMI Advisory Group was formed through an open call for participation, advertised through established M‐RIC service user and carer groups, public advisors and community consultation workshops. Participants were invited to express interest in joining the CMI PPIE Advisory Group. To ensure inclusive selection criteria, we aimed for a combination of carers, service users and community representatives. We ensured diversity in terms of age, gender, ethnicity and socio‐economic background and included individuals with varying engagement experiences to bring fresh and expert perspectives.

From this process, 10 individuals were selected to form the CMI PPIE Advisory Group. The CMI PPIE Advisory Group meets on an ad hoc basis, according to the specific demands arising from various project leads overseeing different components of the system, including Communications, Information Governance, PPIE and Community Engagement. Meetings were scheduled as required, allowing flexibility for the group to provide targeted feedback at critical points in the project timeline. A PPIE research engagement coordinator supported the CMI PPIE Advisory Group's activities to ensure continuity and genuine collaboration among members. All individuals involved with the CMI co‐production activities were reimbursed for their time and travel. Activities ranged from participation in the co‐development of materials, shaping of engagement questions and plans, and their involvement in the CMI Advisory Group. Reimbursement was provided in line with NIHR guidelines to recognise the value of their contributions and to reduce barriers to participation. Funding for this was allocated from M‐RIC, specifically set aside to support PPIE infrastructure. This ensured that appropriate financial support was in place to enable involvement throughout the development of the CMI programme. We could not provide cash advances to individuals due to organisational policy; as a result, individuals could only claim reimbursement after their activities had taken place. This presents a barrier to inclusive participation, particularly for individuals facing financial difficulties. The group collectively established a set of values for involvement through discussion (Table 4).

**Table 4:** CMI Advisory Group values for involvement.

Values	Description	Action
No tokenism	All members share genuine power. We listen and are listened to, and we only participate when confident we understand the processes, ensuring our involvement is meaningful and not merely symbolic.	Ensured all members understood their roles and the impact of their participation and contributions. Building and maintaining relationships.
Fair reimbursement	Service users and carers are fairly compensated for their time and effort through expenses and reimbursement.	Aligned reimbursement policy with NIHR guidelines to ensure timely payment for participants' time and expenses.
All voices are heard	Efforts are made to reach under‐represented groups and individuals. While not all groups have been included yet, their involvement remains a key priority.	Developed an outreach plan to engage under‐represented groups and build trust through engagement.
Plain language	Communication is clear and accessible, recognising that not everyone processes information at the same speed.	Developed plain‐language visual aids in multiple formats, for example, drama role play, to make communication inclusive and easy to understand.
Bottom‐up networking	Consultations start with those who have lived experience of mental and general healthcare services, ensuring their insights shape the process.	Prioritised lived experiences by involving participants in agenda‐setting and decision‐making processes.
Consultation with impact	Consultations must lead to tangible outcomes, with clear timelines and visible results. This helps avoid “consultation fatigue,” where input is sought but no feedback or outcomes are provided.	We provided regular feedback and updates on input received to demonstrate how consultations influence outcomes.

#### 3.5.4 Integration With CMI Core Group

Four representatives from the CMI PPIE Advisory Group were invited to sit on the CMI Core Group. The interested individuals completed an expression of interest form using the PPIE activities form, and one‐to‐one discussions with interested individuals were undertaken to understand their motivations to join the core group. The CMI Core Group meets bi‐weekly, ensuring direct input into high‐level decision‐making around implementation. This direct integration ensured that the perspectives and insights of service users and carers were embedded in the core decision‐making processes of the system. It also provided a formal mechanism for the advisory group's feedback to be channelled into strategic and operational planning.

The CMI Core Group is composed of strategic personnel from Mersey Care NHS Foundation Trust, including key representatives from Communications, Information Governance and PPIE at a senior level. This group plays a key role in influencing change and driving the strategic direction of the CMI system. Their involvement ensures that the system is aligned with organisational priorities and that decision‐making processes reflect both strategic and operational needs. The CMI Core Group reports to the Strategy and Quality Committees within Mersey Care NHS Foundation Trust through the R&I Service, to ensure that the CMI system not only functions effectively but also is in alignment and integrates with the broader strategic intentions of Mersey Care NHS Foundation Trust.

## 4 Discussion

The CMI system facilitates contacting service users for research opportunities for up to 5 years following discharge from a service. Participation in CMI is assumed, with individuals having the option to ‘opt out’ if they do not wish to be contacted for research. They can also ‘opt back in’ easily should they wish to do so. The goal is to ensure that the voices of community members and service users are included in research, facilitating inclusive solutions to local and regional challenges.

The primary purpose of research is often to improve services, develop new interventions and address societal challenges [17]. Traditionally, a top‐down approach has been used to recruit marginalised groups, in which researchers design projects and then recruit participants through methods like posters, healthcare provider referrals, media advertisements and snowball sampling [18]. These traditional methods present barriers to recruitment, such as high reliance on researchers or healthcare providers' judgements about suitable participants [19]. Cultural norms may also discourage individuals from engaging in research [20] as we found during the engagement phase, with a few minoritised ethnic communities either being unfamiliar with health research or simply asking ‘what is research?’. In contrast, the CMI system seeks to normalise research participation by integrating it into routine service use and addressing some of these barriers. By requiring individuals to actively ‘opt out’ rather than ‘opt in’, the system aims to reduce obstacles that traditionally exclude diverse populations from research.

Research into mental health has consistently highlighted issues of exclusivity [21, 22]. Recommendations to enhance inclusivity often involve targeted strategies, such as training to support LGBTQ+ participation [23], building trust in underserved populations [24], or using multimedia to engage individuals with mental health challenges [22]. However, these approaches can be resource‐intensive and challenging to implement, especially for healthcare workers who often lack the time for research‐related activities [19].

The CMI system makes service users visible for research opportunities as a routine part of service provision. This automatic inclusion approach ensures that underserved populations are always considered for participation (unless the opt‐out) without placing additional burden on clinicians. It provides a standardised method to foster a more inclusive research environment and demonstrates to potential participants that their contributions are valued in shaping the future of research and care.

The CMI system, as adapted and implemented for Mersey Care NHS Foundation Trust, is a transformative system designed to make healthcare research more accessible and inclusive. Co‐developed in response to themes identified in stakeholder workshops (e.g., data protection, patient and public engagement, and effective communication), our CMI approach would help address barriers that have traditionally limited participation in mental health research.

By shifting from an ‘opt‐in’ to an ‘opt‐out’ model, the system simplifies participation while safeguarding patient privacy and autonomy through robust data protection measures. Collaboration with service users, carers and communities ensures that the system reflects diverse needs and lived experiences. This paper describes how stakeholder feedback has been incorporated into the design and implementation of CMI, focusing on its commitment to transparency, inclusivity and trust.

### 4.1 Data Protection: Ensuring Transparency and Safeguarding Privacy

We acknowledge stakeholder concerns regarding transparency in the CMI system and reaffirm our commitment to clear, accountable practices. The CMI system connects service users with research opportunities while respecting their communication preferences and privacy. Automatic enrolment ensures inclusivity; however, opting out remains straightforward via an online form, which was designed with service users for usability, supported either by the CMI Team or through their clinical team. These options are prominently outlined on the Mersey Care NHS Foundation Trust website (See Supplementary Materials) and in public materials, with user preferences regularly updated in the Trust's electronic Master Service users Index (MSI) to maintain accurate capture of service users' opt‐in/opt‐out decisions.

Robust safeguards underpin CMI's transparency and data protection commitments. Service users who ‘opt out’ through the CMI system or the NHS National Data ‘Opt‐Out’ are excluded from research communications, and their preferences are updated frequently. Data queries, for example, to identify service users who might be eligible for a given research study, undergo rigorous privacy checks, including the use of statistical disclosure controls for queries resulting in small numbers of eligible participants that might inadvertently identify individuals. Moreover, all research using the CMI system must be approved by an institutional Research Ethics Committee (REC) or the Health Regulatory Authority (HRA).

Stakeholder consultations highlighted the importance of embedding patient and carer voices into the programme's design. In response, CMI has prioritised PPIE, ensuring service users and carers actively shape the initiative. Collaborative workshops and feedback sessions have informed key features, such as clear ‘opt‐out’ processes and accessible communication materials, ensuring CMI reflects the needs of its diverse user base.

Creative engagement activities, such as the CMI Drama Project, emerged in response to feedback requesting innovative ways to convey the value of research. This approach uses storytelling to engage underserved communities and address barriers such as language or cultural differences. Furthermore, dedicated PPIE leads ensure lived experiences directly influence the system's development, building trust and inclusivity.

### 4.2 Communication Activities: Enhancing Outreach and Accessibility

Effective communication was identified as a key theme during workshops, with stakeholders stressing the need for clear, inclusive and accessible messaging. In response, CMI has adopted a multichannel communication strategy to reach diverse audiences. Internally, Mersey Care NHS Foundation Trust staff are informed and updated on CMI through weekly newsletters, the intranet and team briefings, equipping them to support and promote the initiative. We also conducted both in‐person and online information sessions. The online session was recorded and added to the staff intranet as a future education tool.

Externally, information was added to patient letter templates and patient screens in waiting areas and sent directly to Mersey Care NHS Foundation Trust members and governors. CMI has also been promoted via Mersey Care and the M‐RIC communication channels such as websites, social media, public email bulletins and the Trust's magazine. A communications toolkit for third sector organisations was produced and distributed widely so they could easily communicate about CMI to maximise the reach to those who might not routinely access Trust resources. This includes useful materials for organisations to add to their communications channels, such as a social media graphic and text, a poster, and short and long versions of newsletter articles.

To supplement these approaches and to ensure a wider audience across Cheshire and Merseyside was reached, we ran a 2‐week local radio advertising campaign (see Supporting Information for transcript). These radio stations have a combined listenership of 500,000 per week. In terms of communicating CMI in different languages, we plan to collaborate with partner organisations to deliver messages in video format using local trusted voices in communities. This was a model used successfully by NHS organisations during the Covid pandemic to deliver messages locally. We established a CMI Champions Programme to mobilise staff, volunteers and service users to advocate for the initiative and foster a grassroots movement that supports participation and ownership of this new model of research participation.

This implementation‐focused account of CMI is rooted in a constructivist approach that prioritises relational, co‐produced knowledge. While we did not collect identifiable demographic data intentionally, to support trust and inclusion, marginalised groups directly shaped key system elements, including communications, opt‐out messaging and design approvals. Their input is now embedded in governance through strategic roles and reporting to the Trust's Quality Committee. Accessibility has also progressed: we have co‐produced an Easy Read version, enabled website translation, and are developing community‐language videos. These efforts are supported by a phased communication strategy aimed at sustaining equity and reach.

### 4.3 Future Work

In this paper, we have described the process of co‐creating CMI to adapt the ‘opt‐out’ framework to local needs, cultures and practices. The next phase of the CMI implementation will focus on scaling and translating its impact through shared learning and collaboration with Oxford Health NHS Foundation Trust. Key priorities include staff training to enhance engagement with diverse groups, addressing language barriers and providing culturally sensitive communication. For CMI to be effective, we require technology to support the querying of CMI participants' data to identify candidate participants relevant to a set of eligibility criteria. If this technology results in queries or ‘filters’ that are sensitive but non‐specific, many patients identified for contact will be false positives (for the eligibility criteria).

Excessive false positives incur resource cost (for contacting patients) who will transpire to be ineligible, and in turn, this risks service users becoming fatigued and frustrated by frequent contact about irrelevant participation opportunities. However, to improve the specificity of queries requires more detailed data (about the service user) to be available, which in turn, represents a compromise between the utility of the CMI system and adhering to data governance legislation (e.g., the principle of data minimisation). Summary data (such as diagnostic codes and age) alongside a persons' CMI preferences will need to be bolstered with further ‘deep’ queries on the service users' source electronic health record to establish more granular descriptions of how a given person matches the eligibility criteria. This raises as‐yet unanswered questions about the legitimate reuse of routinely collected clinical data for research purposes. For example, understanding how a service user would respond to a contact through CMI, where algorithmic mining of their electronic health record examines aspects of historical abuse or trauma to determine eligibility. There is unlikely to be a single uniform approach that guarantees acceptability, but consistent with the approach taken in Phases 1 and 2 of our CMI implementation, co‐producing these processes and technologies will be vital.

### 4.4 Strengths and Limitations

We made a concerted effort to reach under‐represented groups during the co‐production phase. This included targeted outreach to communities with historically lower levels of research engagement, such as people with learning disabilities, older adults, minoritised ethnic communities (including asylum seekers) and individuals who are digitally excluded or who have lived experience of mental ill health. To support this, we developed an outreach communication plan using multiple channels, including workshops, digital media and community outreach to ensure clarity and transparency. This proactive communication approach strengthened patient and public trust, reassuring participants about data protection and research participation. However, co‐production was resource‐intensive, posing challenges for scalability and consistency across NHS Trusts. Despite efforts to enhance transparency, ongoing communication is needed to address data protection concerns and sustain trust. It is important to acknowledge that not all voices have yet been included; engaging with diverse communities remains an ongoing and central priority for both M‐RIC and CMI. As part of this, we are exploring how to embed health inequalities impact monitoring more formally in future engagement activities, to continuously monitor who is participating and who may still be missing.

## 5 Conclusion

The need for inclusive, accessible health research has never been more urgent, particularly in addressing the inequalities inherent in accessing mental health services. The CMI system exemplifies how innovative, co‐produced initiatives can tackle traditional barriers to participation, fostering trust and equity in research. By integrating participation into routine care and involving stakeholders at every stage of development, CMI provides a scalable model for embedding inclusivity into the research process. This approach not only advances knowledge but also empowers individuals and communities to have a voice in shaping research priorities and outcomes. The CMI approach also presents the opportunity for broader application beyond formal research settings. Its principles, such as equitable partnerships, shared decision‐making and valuing lived experience, sit closely with elements of effective service transformation and system redesign. As healthcare systems continue to evolve, initiatives like CMI highlight the importance of aligning research with the principles of inclusivity, transparency and shared decision‐making to ensure that no one is left behind in the pursuit of better health.

## Author Contributions

**Oladayo Bifarin:** conceptualisation, methodology, data curation, writing – original draft, formal analysis, visualisation, resources, writing – review and editing, supervision, funding acquisition. **Jahanara Miah:** data curation, formal analysis, visualisation, writing – original draft, project administration. **Michelle Harvey:** resources, project administration, writing – review and editing. **Gail Faragher:** project administration, writing – review and editing. **Jade Thai:** writing – review and editing, resources. **Dennis Dewar:** resources, writing – review and editing. **Jenny Garden:** resources, writing – review and editing, project administration. **Lindsey Nicholson:** project administration, writing – review and editing, resources. **Nicola Wilson:** funding acquisition, project administration, writing – review and editing, resources. **Nusrat Husain:** writing – review and editing, conceptualisation. **Dan W. Joyce:** conceptualisation, writing – review and editing, resources, funding acquisition, writing – original draft.

## Conflicts of Interest

Oladayo Bifarin is a National Institute for Health and Care Research Leader. The views expressed in this article are those of the author(s) and not necessarily those of NIHR or the Department of Health and Social Care. Nusrat Husain has previously served as a Trustee for the Abaseen Foundation UK, Pakistan Institute of Living and Learning, Lancashire Mind UK, and Manchester Global Foundation. N.H. is also an executive member of the Academic Faculty at the Royal College of Psychiatrists, London, and an NIHR Senior Investigator.

## Supporting information

Supporting materials for Count Me In paper.

## Data Availability

The authors have nothing to report.
