# Supplementary material for: Co‐Production and Implementation of ‘Count Me In’: A Bottom‐Up Approach to Inclusive Research and Participation in a National Health Service in England
Source: Health Expect. 2025 Jun 23;28(3):e70326. doi: 10.1111/hex.70326 (PMC12183467; doi:10.1111/hex.70326)

**Supplementary Materials**

**
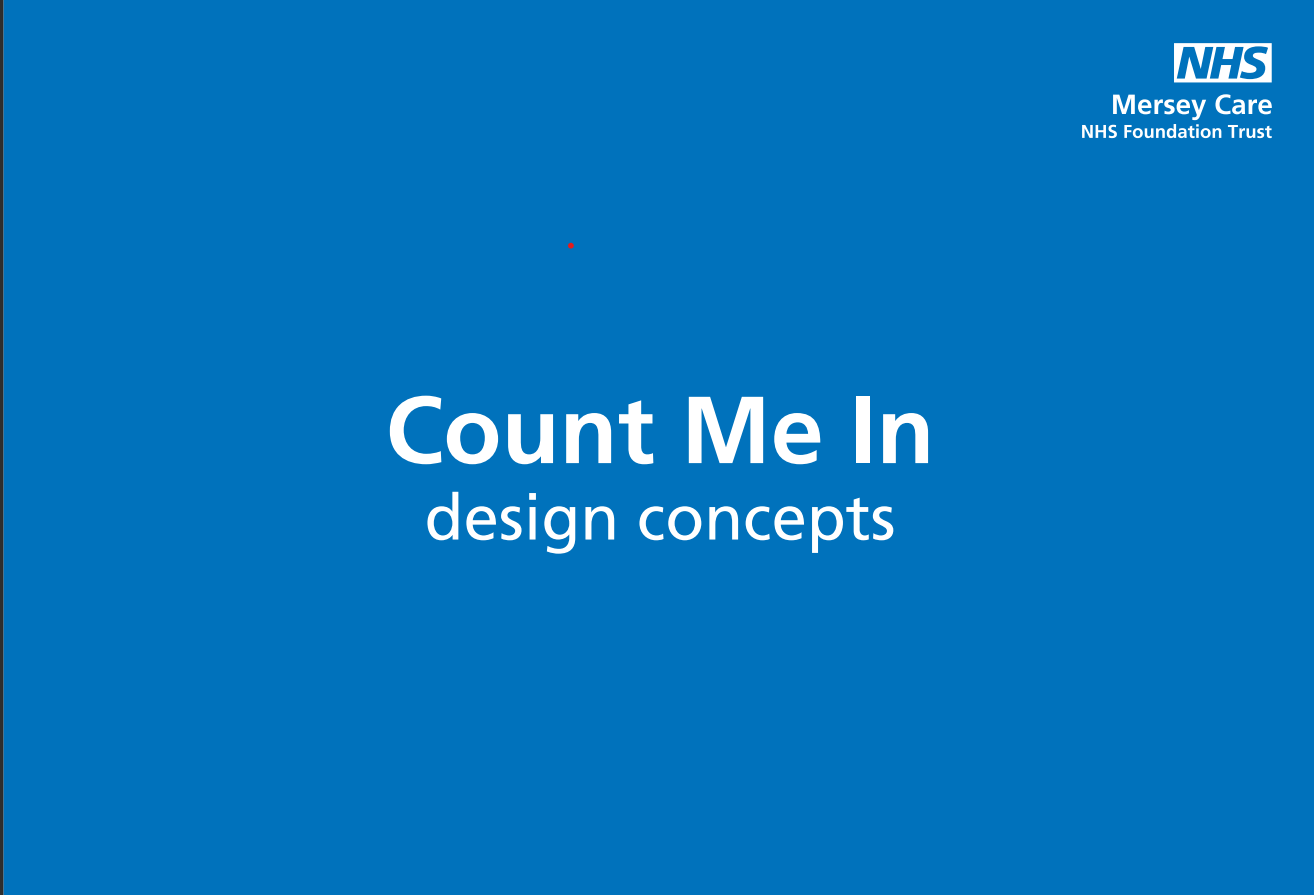
Supplementary Material 1: Count Me In design concepts**

**
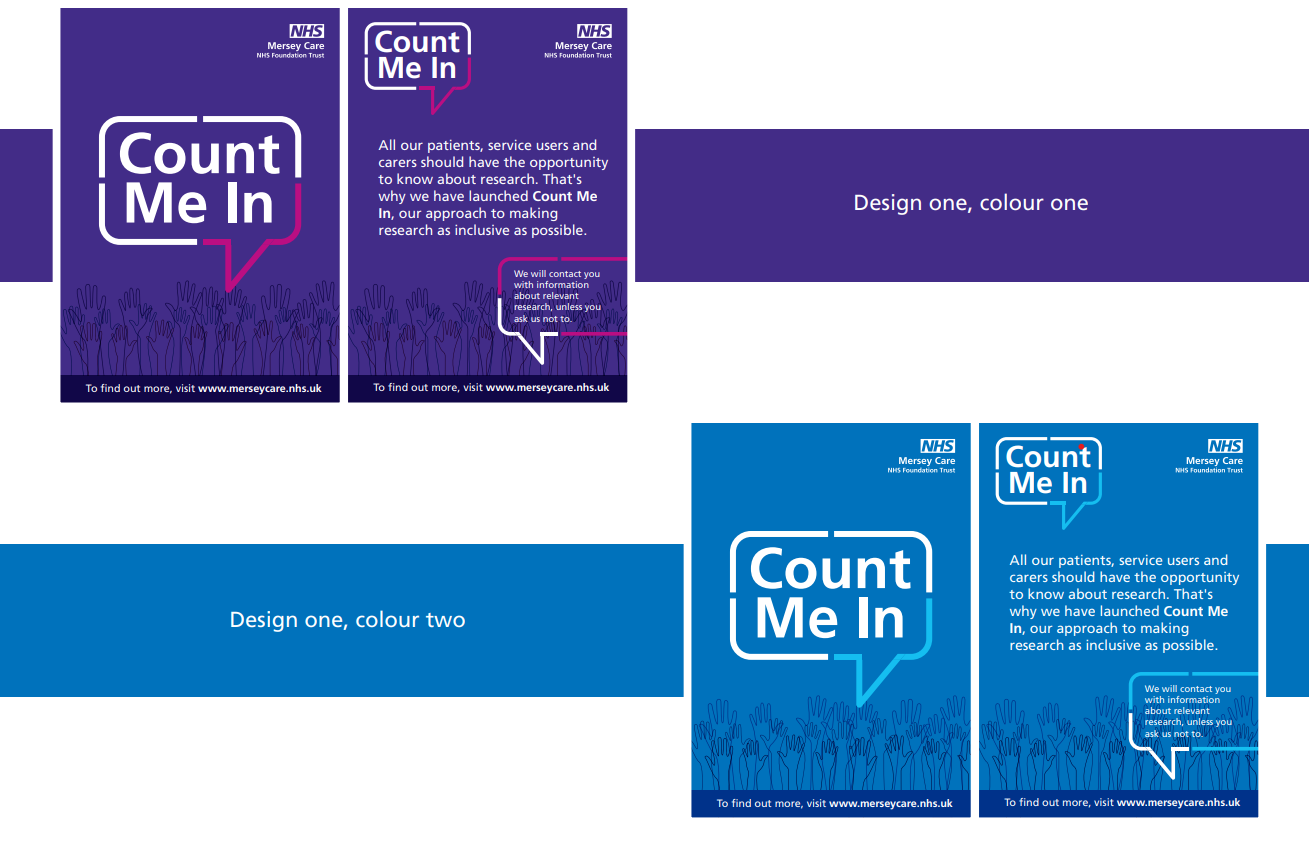
**


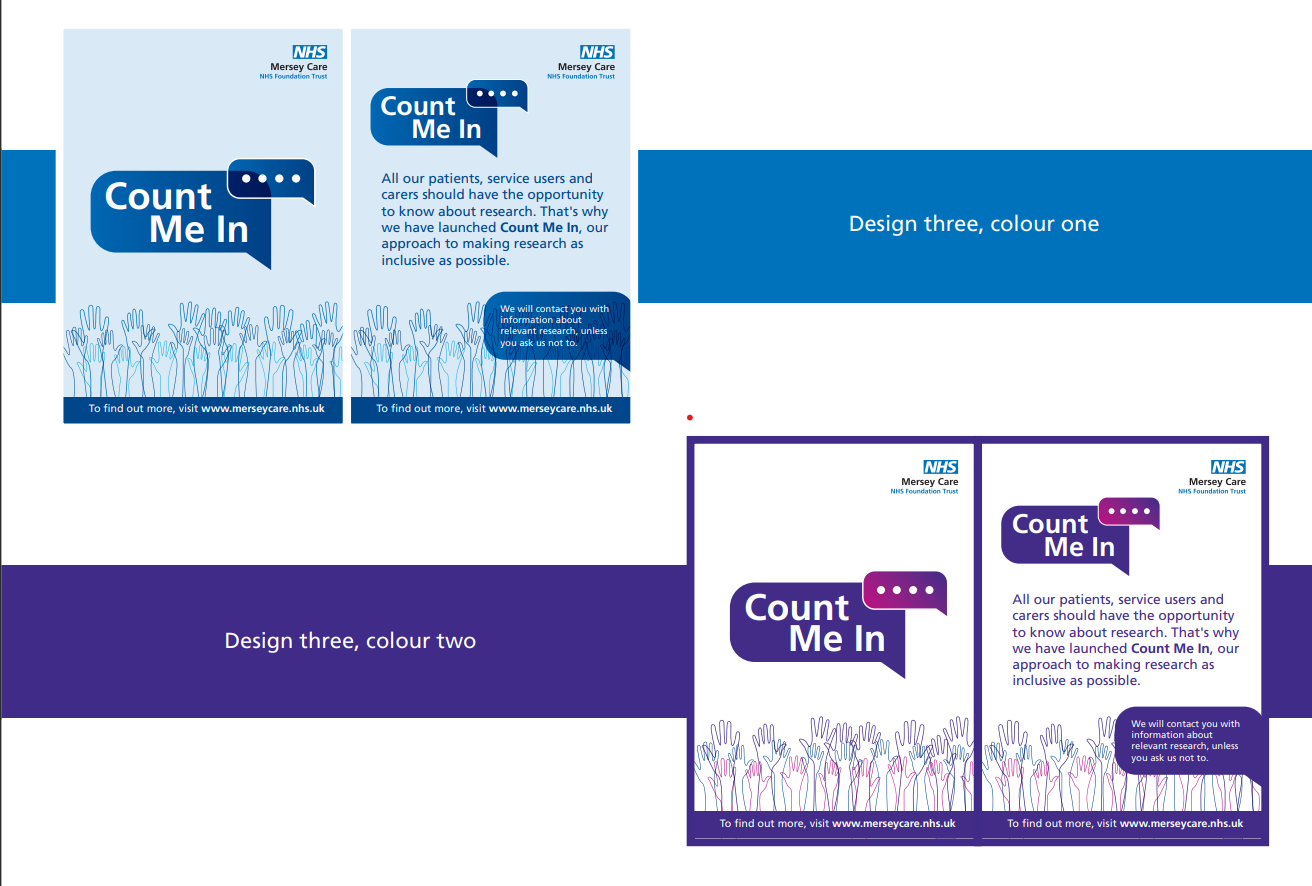
**
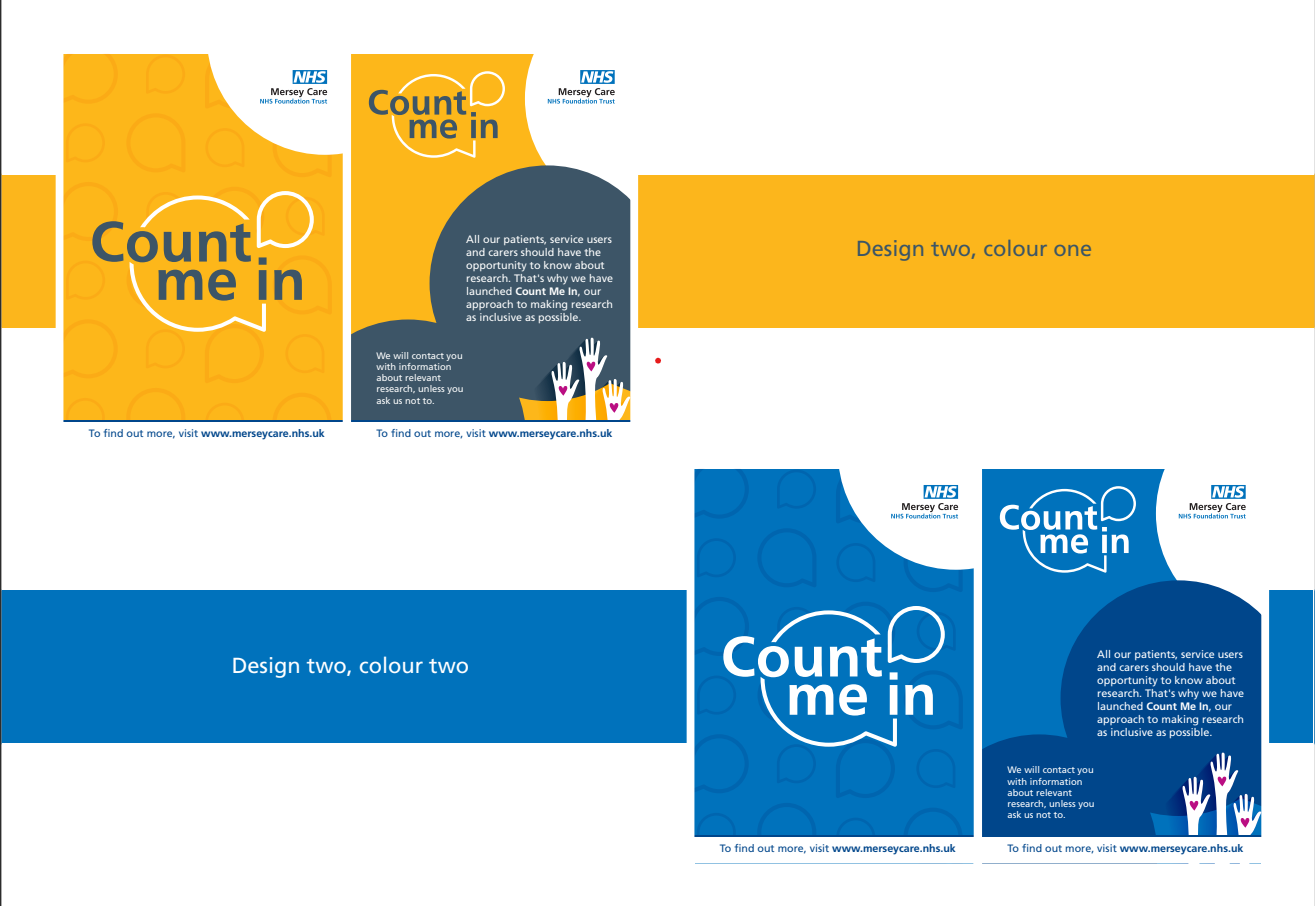
**

**
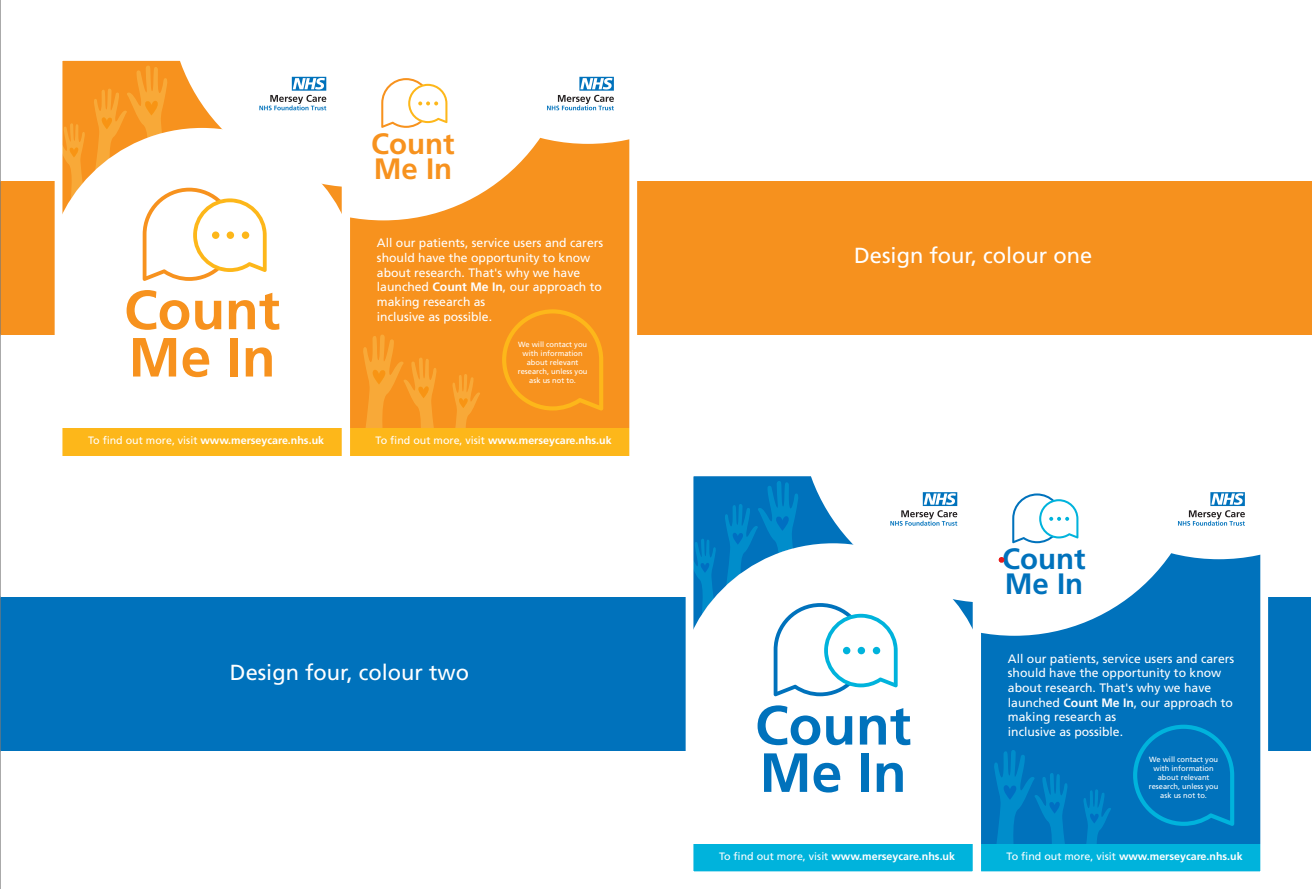
**

**
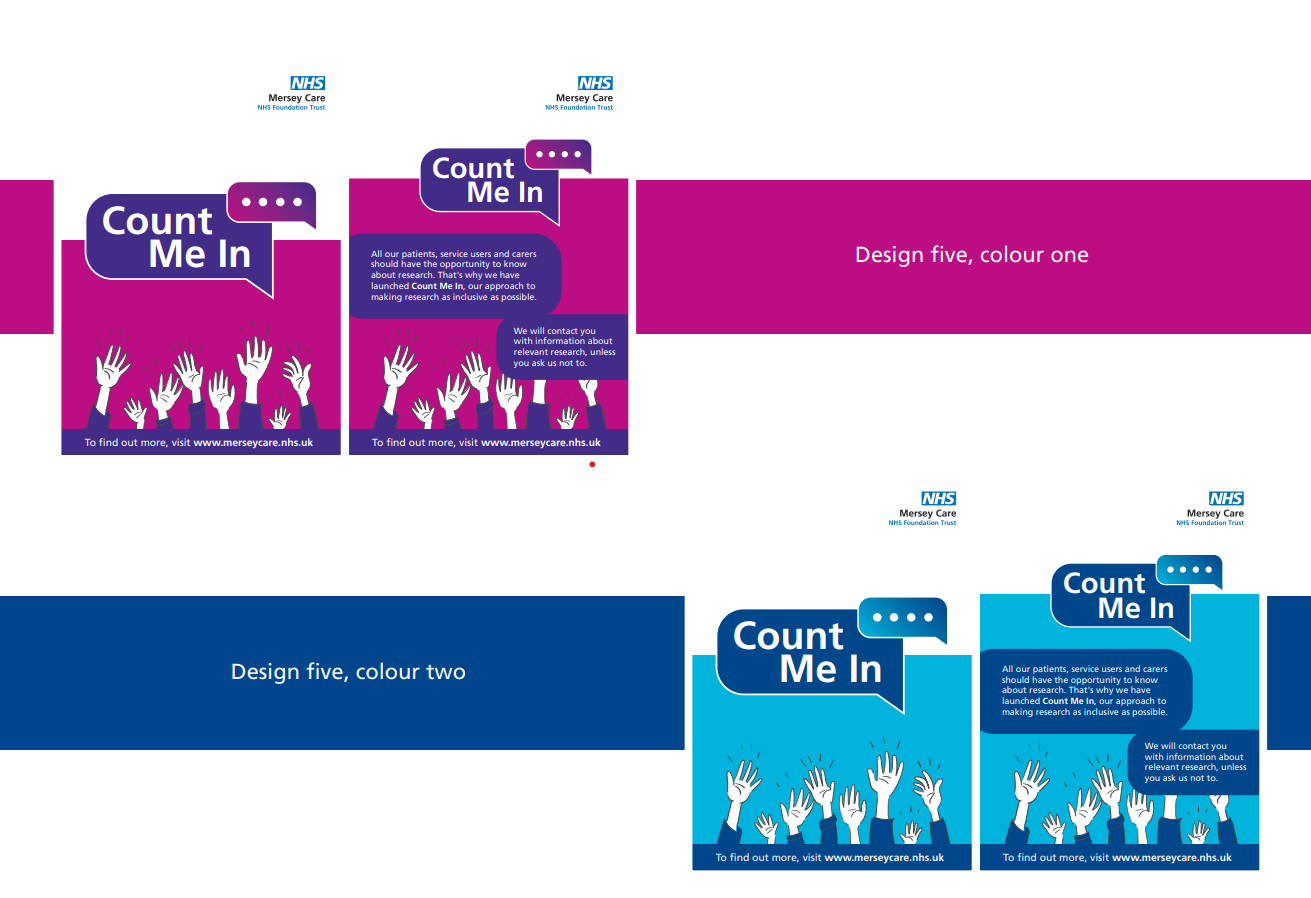
**


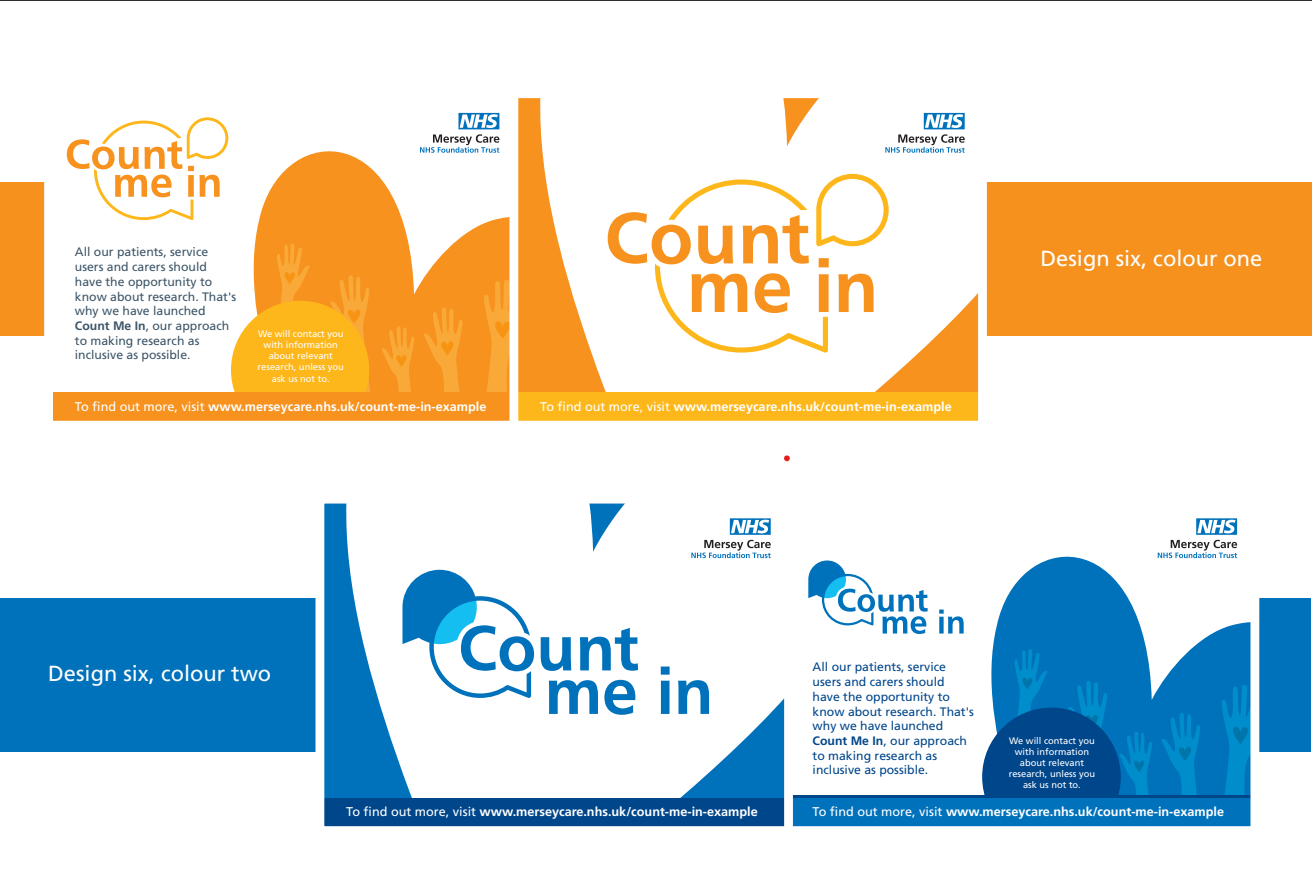


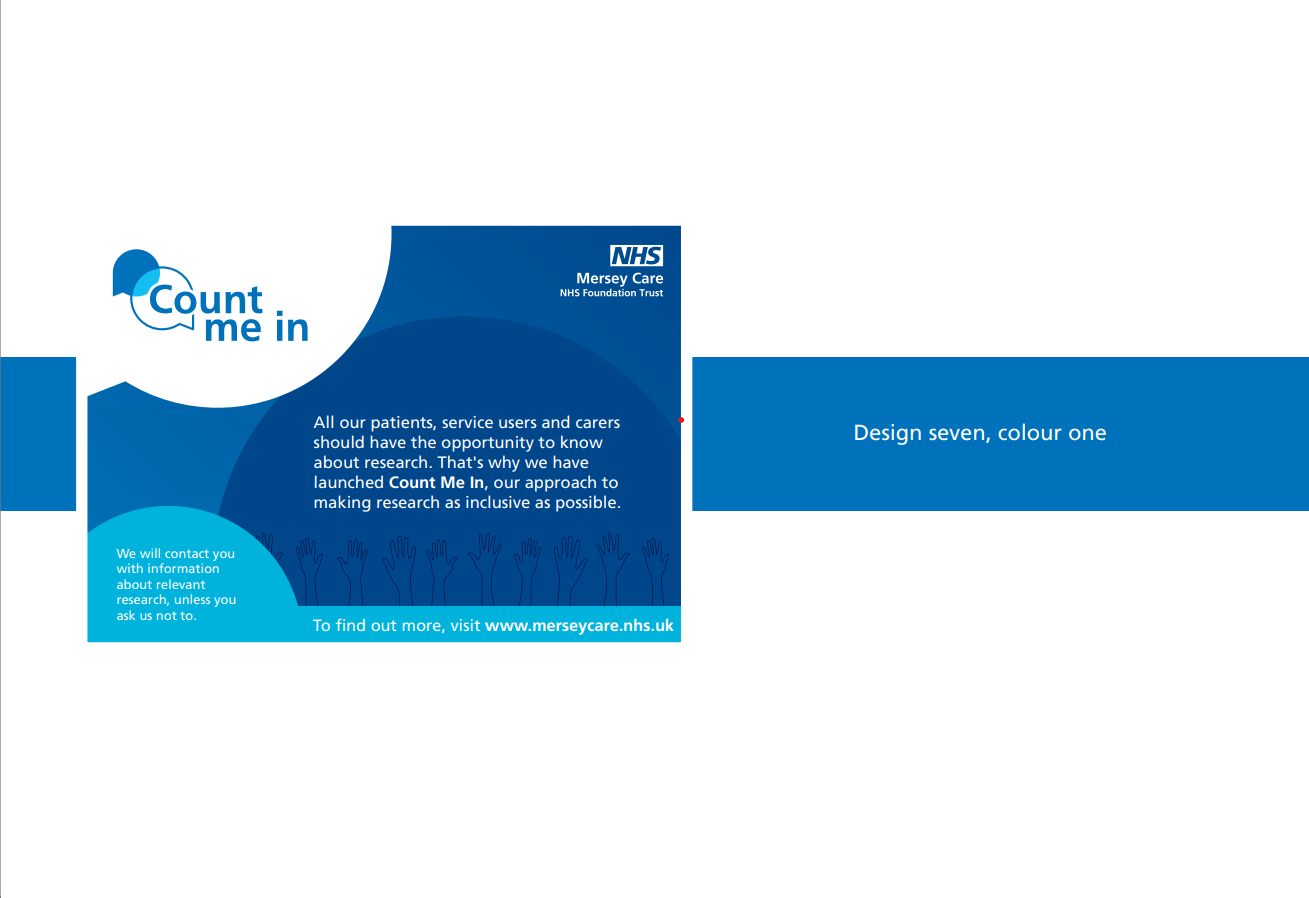


**Supplementary Material 2: Count Me In final design Toolkit**

Poster:


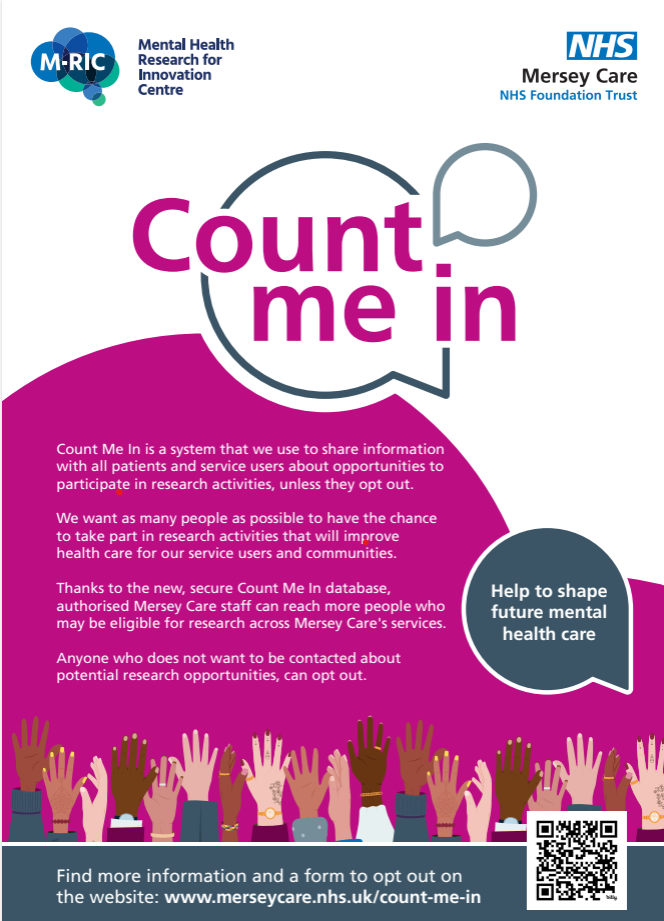


Social media tile:

**
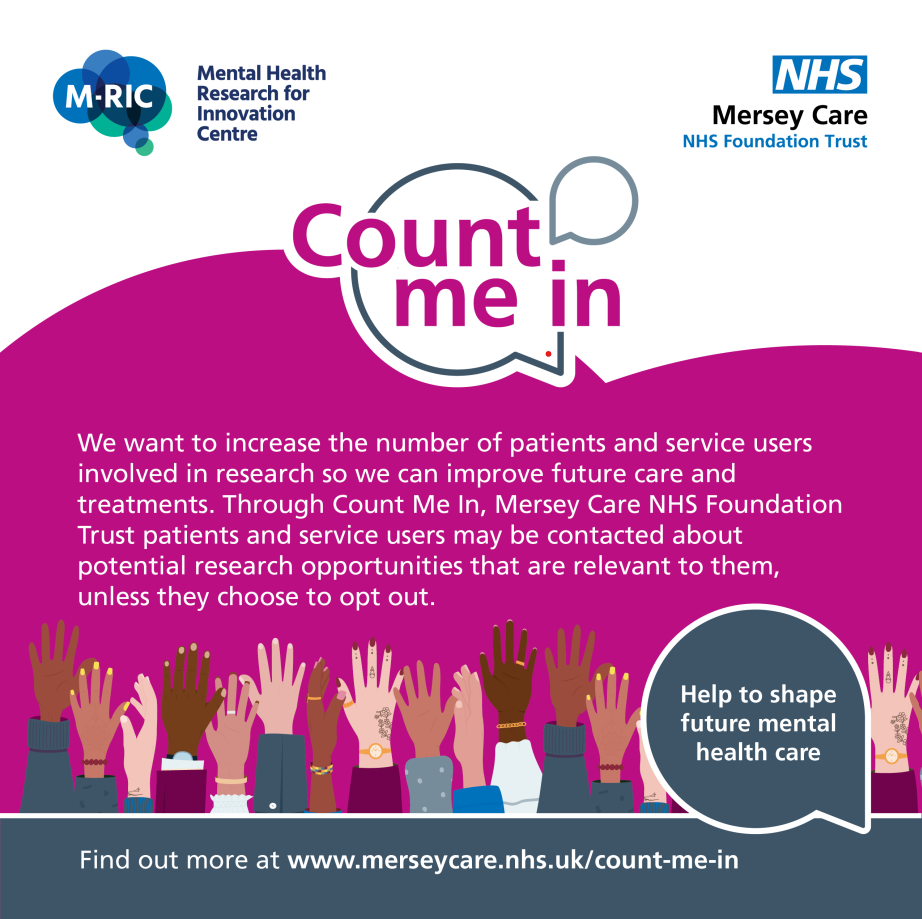
**

**Supplementary Material 3: Count Me In Radio Advert**

Listen here: <https://mric.uk/clinicians-and-service-users-unite-to-champion-count-me-in/>

Transcript:

Research helps improve healthcare for you and your family.

That’s why Mersey Care and the Mental Health Research for Innovation Centre are rolling out Count Me In.

If you’re a Mersey Care service user, you’ll be counted in to hear about research opportunities that are relevant to you.

There’s no pressure to take part and you can opt out at any time.

But by supporting research you could make a real difference.

Visit merseycare.nhs.uk and search Count Me In today.

**Supplementary Material 4: Count Me In webpage**

[www.merseycare.nhs.uk/count-me-in](https://www.merseycare.nhs.uk/count-me-in)


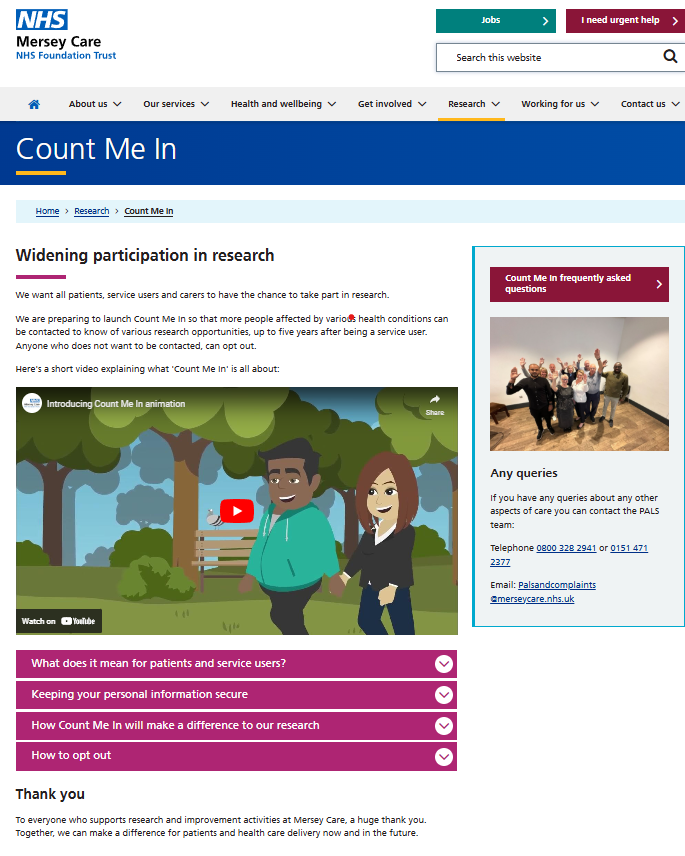

Supplement: Supplementary file 1 — Supporting materials for Count Me In paper. [file HEX-28-e70326-s001.docx]
